# A160 A SARCOMATOID CARCINOMA OF THE COMMON BILE DUCT PRESENTING AS PAINLESS JAUNDICE AND EXTRAHEPATIC BILIARY OBSTRUCTION

**DOI:** 10.1093/jcag/gwac036.160

**Published:** 2023-03-07

**Authors:** R Chan, A Kohansal, M Stewart

**Affiliations:** Gastroenterology, Dalhousie University, Halifax, Canada

## Abstract

**Background:**

Sarcomatoid carcinomas are extremely rare tumors made of epithelial and mesenchymal elements. They have been found in various organs, but presence in the common bile duct (CBD) has only been reported a handful of times. Insight regarding the clinical history, histopathology, treatment, and prognosis is limited. The majority of CBD sarcomatoid carcinomas have occurred in elderly women, including this case. Surgical resection is the mainstay of treatment and the roles for chemotherapy and radiation therapy are undetermined. Prognosis is variable, but generally poor.

**Purpose:**

Additional information regarding sarcomatoid carcinomas of the CBD will aid in establishing a timely diagnosis and may alter treatment options and prognosis. We aim to add to the limited literature surrounding this rare CBD neoplasm.

**Method:**

A 71-year-old female presented with painless jaundice, decreased appetite, and weight loss. Initial investigations showed an alkaline phosphatase (ALP) of 3075 U/L, aspartate transaminase (AST) of 507 U/L, alanine aminotransferase (ALT) of 298 U/L, total bilirubin of 325.5 µmol/L, and direct bilirubin of 254.1 µmol/L. Initial computed tomography (CT) scan done showed marked intra- and extrahepatic biliary ductal dilation with appropriate tapering and the presence of a distal CBD hyperdensity. Tissue biopsy obtained by endoscopic retrograde cholangiopancreatography (ERCP) was suspicious for a malignant peripheral nerve sheath tumor. The differential also included synovial sarcoma and sarcomatoid mesothelioma.

**Result(s):**

After multidisciplinary discussion involving hepatobiliary surgery, medical oncology, and radiation oncology, pancreaticoduodenectomy was performed; there was no role for neoadjuvant/adjuvant chemotherapy/radiation therapy. Final pathology revealed a well circumscribed mass with a narrow attachment to the posterior CBD, measuring 5.6 x 3.2 x 2.8 cm. Immunohistochemistry showed mixed differentiation with sarcomatoid, squamous, and glandular components, consistent with a sarcomatoid carcinoma. H3K27me3 expression was lost in neoplastic cells. Immunostaining showed strong expression of vimentin and weak expression of CD34, calretinin, CK5 and EMA. Post-operative course was complicated by pancreaticojejunal leak, surgical wound infection, myocardial injury, and esophageal stricturing. 14-weeks post-pancreaticoduodenectomy the patient was found to have *C.difficile* infection and a perforated viscus, exact location of which was not visible on imaging. Emergent laparotomy revealed a gastroduodenal leak and diffuse small bowel ischemia. The patient passed away shortly after emergent surgery.

**Conclusion(s):**

This case illustrates a rare presentation of sarcomatoid carcinoma within the CBD and highlights some of the diagnostic challenges, limited management strategies, complications, and potential poor prognosis of the disease. Further research is required to guide diagnosis and management, and ultimately improve patient outcomes.

**Please acknowledge all funding agencies by checking the applicable boxes below:**

None

**Disclosure of Interest:**

None Declared

